# Erdheim-Chester Disease: A Case Report Exploring Multisystemic Involvement

**DOI:** 10.7759/cureus.74853

**Published:** 2024-11-30

**Authors:** Francisco Antunes, Rúben Maia, Sara Henriques, Ana Ribeiro, Francisca Guimarães

**Affiliations:** 1 Radiology, Unidade Local de Saúde Vila Nova de Gaia | Espinho, Vila Nova de Gaia, PRT; 2 Neuroradiology, Unidade Local de Saúde Vila Nova de Gaia | Espinho, Vila Nova de Gaia, PRT; 3 Internal Medicine, Unidade Local de Saúde Vila Nova de Gaia | Espinho, Vila Nova de Gaia, PRT; 4 Pathology, Unidade Local de Saúde Vila Nova de Gaia | Espinho, Vila Nova de Gaia, PRT

**Keywords:** 18f-fdg pet/ct, erdheim-chester, genetics, neurology, oncology, radiology

## Abstract

Erdheim-Chester disease (ECD) is a rare, multisystemic, non-Langerhans cell histiocytic neoplasm predominantly affecting middle-aged males in their fifth to seventh decades of life. It often presents with nonspecific symptoms, leading to a delay in its diagnosis. We report a case of an 85-year-old male with multisystemic manifestations, including retroperitoneal, skeletal, vascular, cardiac, orbital, and central nervous system (CNS) involvement. Imaging revealed characteristic findings such as bilateral osteosclerosis, perirenal infiltration (hairy kidney sign), and dural-based intracranial masses. Histopathological analysis confirmed the diagnosis, identifying CD68+ histiocytes and the BRAFV600E mutation. The patient was managed initially by Internal Medicine and later referred to Hemato-Oncology for further treatment. This report highlights the importance of maintaining a high index of suspicion for this rare disorder, as well as adopting a multidisciplinary approach toward its treatment, integrating clinical, radiological, and histopathological data.

## Introduction

Erdheim-Chester disease (ECD) is a rare, multisystemic, non-Langerhans cell histiocytic neoplasm marked by the infiltration of tissue with lipid-laden cytoplasmic inclusions. It predominantly affects adult males in their fifth-seventh decades of life and has a typically insidious onset, which can cause a delay in its diagnosis [1]. Skeletal involvement, especially symmetrical osteosclerosis in the metaphysis of long bones, is nearly pathognomonic, leading to bone pain in most patients [2,3]. Central nervous system (CNS) and cardiovascular manifestations are associated with a poorer prognosis [1,4]. Other sites of infiltration include the perivascular space, the retroperitoneum (i.e., around the kidneys), and pleuropulmonary tissue [1].

Recent advancements in understanding the disease's molecular pathogenesis have led to the reclassification of ECD as a histiocytic neoplasm, rather than a purely inflammatory condition [1], as described in the latest 2021 WHO classification of CNS tumors [5]. The recent findings of BRAFV600E mutations, present in more than half of ECD cases, have validated this, providing evidence of both clonal proliferation (BRAFV600E mutation-associated) and non-clonal accumulation of histiocytes (related to the inflammatory cascade) with implications for targeted therapy [6].

Imaging is essential for diagnosing ECD, as it aids in determining the extent of organ involvement and possibly guiding biopsy and therapy decisions, although histopathological confirmation remains essential [2,7]. Owing to its multisystemic nature, an interdisciplinary approach with imaging and histological and clinical data is crucial [8,9]. Advances in treatment, including targeted therapies like vemurafenib for patients with BRAF mutations, have led to better outcomes, even though the disease remains associated with significant morbidity and mortality [10,11].

## Case presentation

Clinical history and initial management

The patient was an 85-year-old male with a previous history of ischemic heart disease and intracranial lesions under investigation for suspected meningiomas. He had been referred to an Internal Medicine outpatient consultation in our hospital, due to nonspecific symptoms including a 15% weight loss, marked asthenia, imbalance, lower limb edema, and weakness for over seven months, as well as polyuria and polydipsia with routine blood tests revealing persistent hypernatremia. Due to these symptoms, a CT of the abdomen and pelvis was performed to exclude a neoplastic process, as well as an MRI of the brain to further characterize the previously depicted lesions and due to clinical suspicion of central diabetes insipidus.

Imaging findings

**Figure 1:**
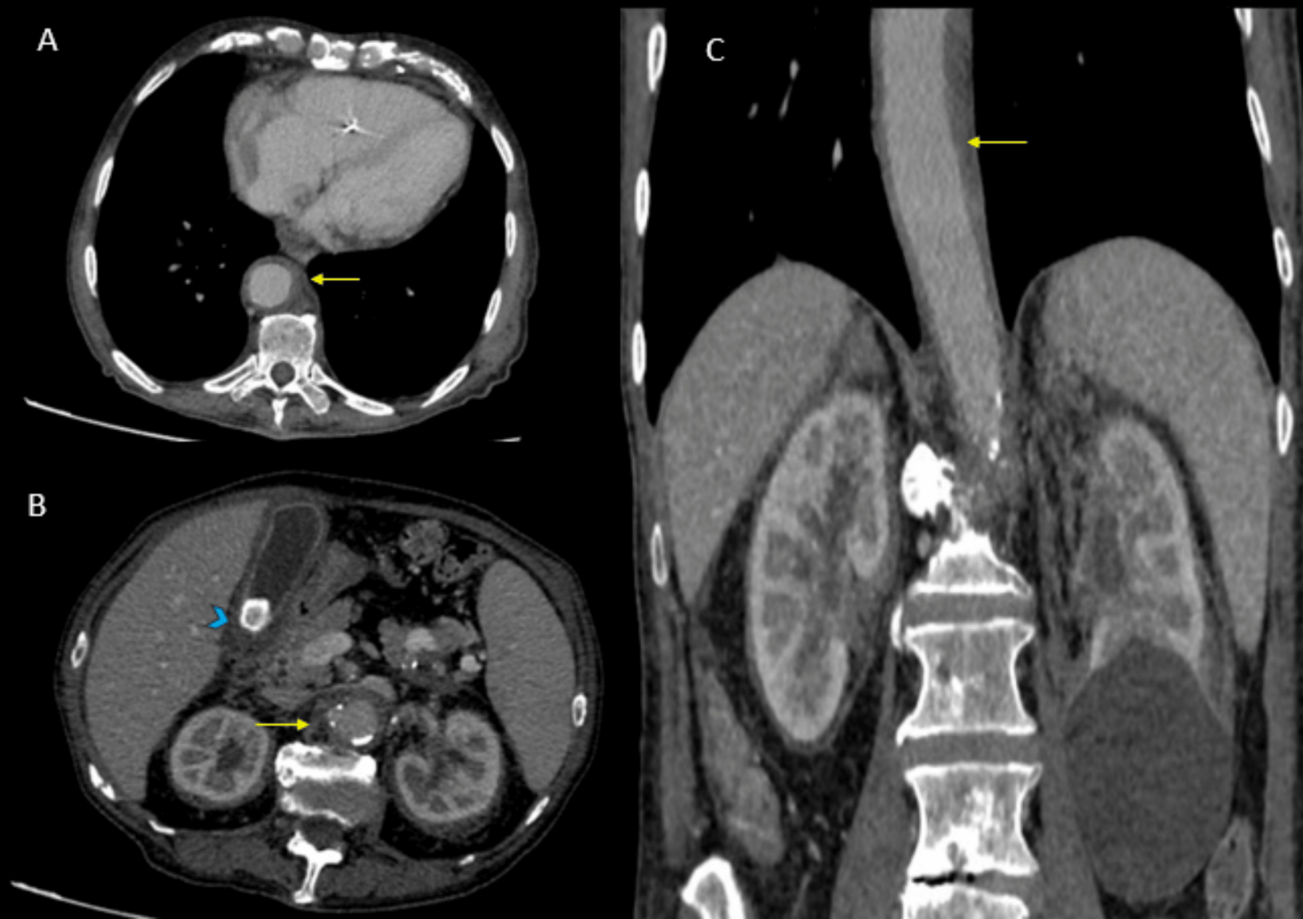
Axial CT of the lower thorax (A) and abdomen (B) shows circumferential coating of the aorta that does not spare the posterior wall (yellow arrows). Coronal reformation (C) also depicts the extent of the tissue infiltration around the aorta (yellow arrow). Incidental gallbladder stones are also seen in B (blue arrowhead) CT: computed tomography

**Figure 2:**
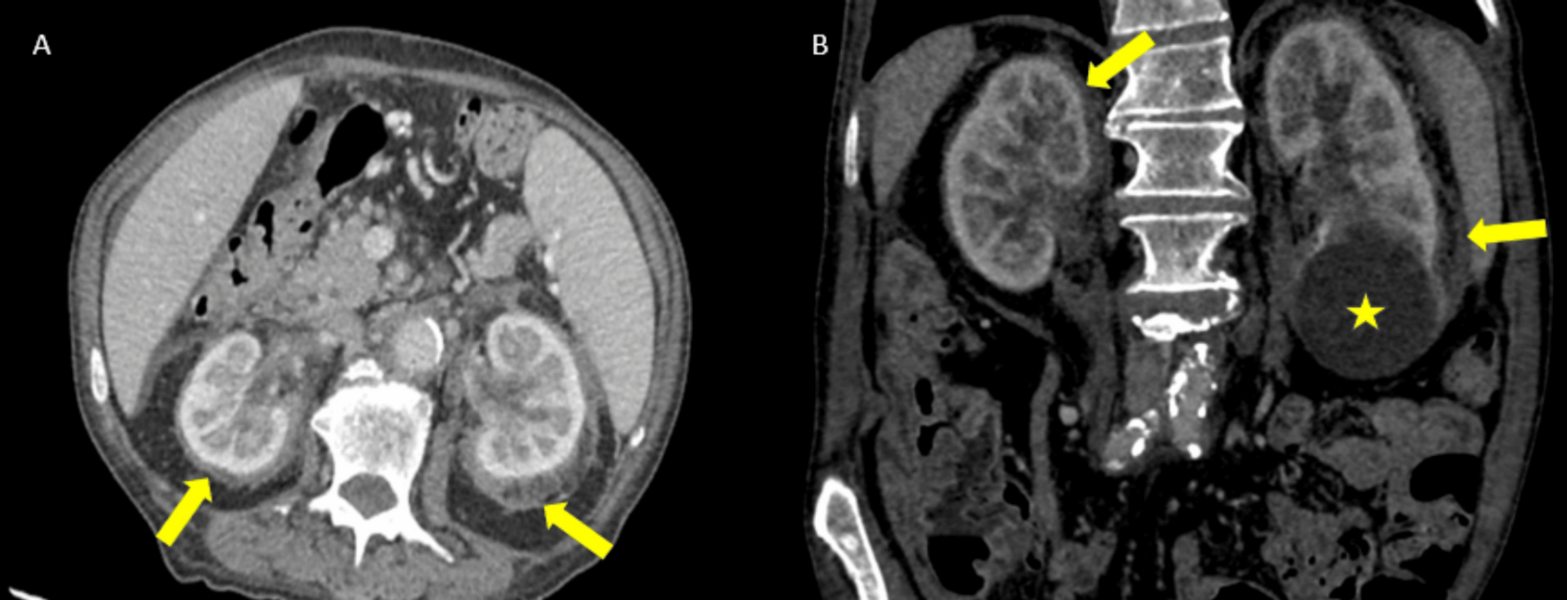
Axial CT (A) and coronal reformation depicts bilateral infiltration of the perirenal fascia – hairy kidney sign (yellow arrows). Large simple cortical cyst is also seen in the lower pole of the left kidney (yellow star) CT: computed tomography

**Figure 3:**
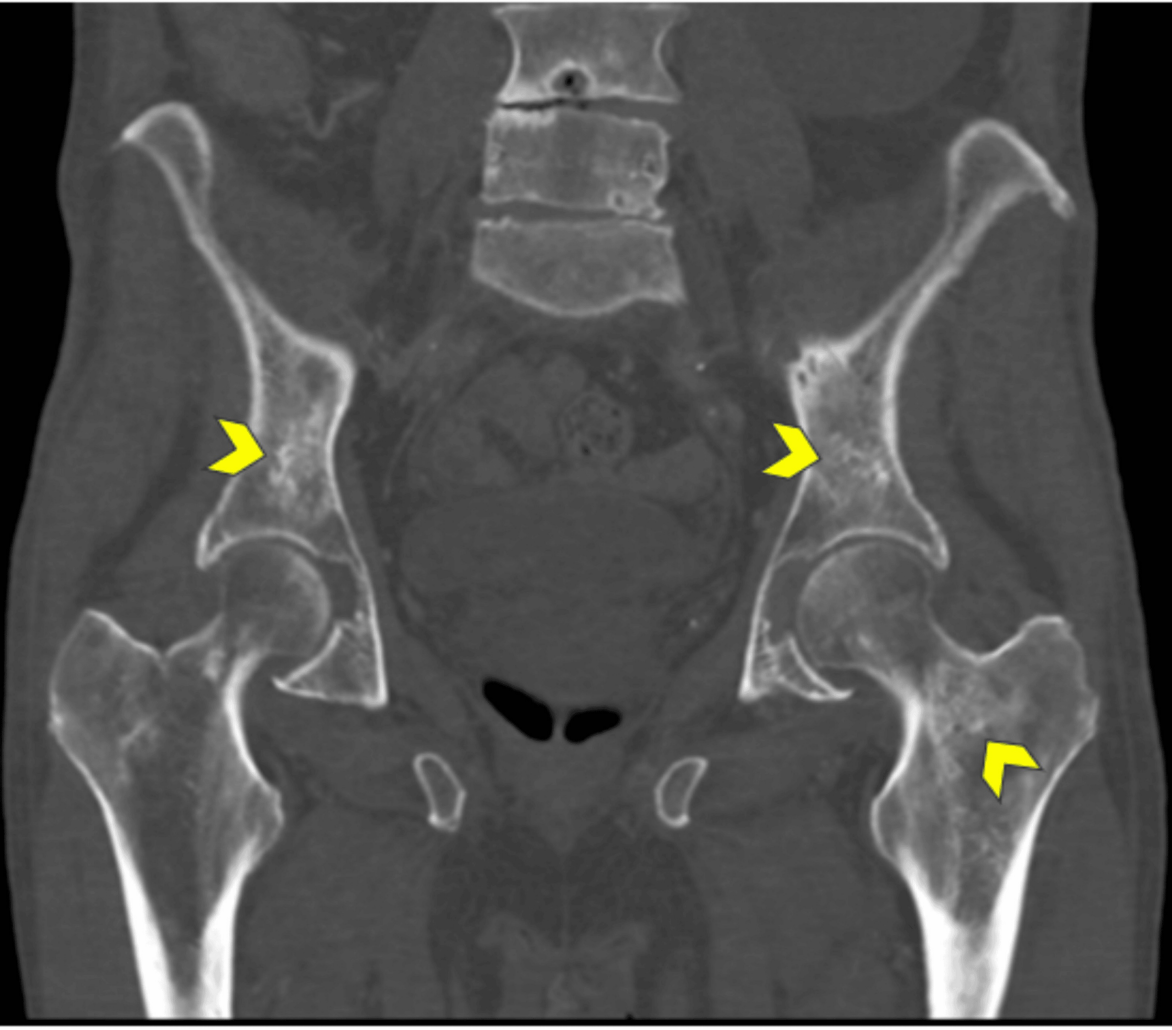
CT coronal reformation on bone window demonstrates small foci of osteosclerosis in both iliac wings as well in the femoral neck/proximal femoral metaphysis (yellow arrowheads). Degenerative changes of the lumbar spine are also seen CT: computed tomography

Abdominal and pelvic CT revealed a circumferential wall coating/thickening of the abdominal aorta and symmetric and bilateral infiltration of the perirenal fascia (hairy kidney sign) (Figures 1, 2). In the bone window, small foci of osteosclerosis were documented in both iliac wings, ilio-pubic rami, and the femoral neck and proximal femoral metaphysis (Figure 3).

Previous head CTs performed in the ER setting for incidental falls had demonstrated a parafalcine intracranial space-occupying lesion initially suggestive of meningioma and an intraorbital mass, showing progressive growth for over a year.

**Figure 4:**
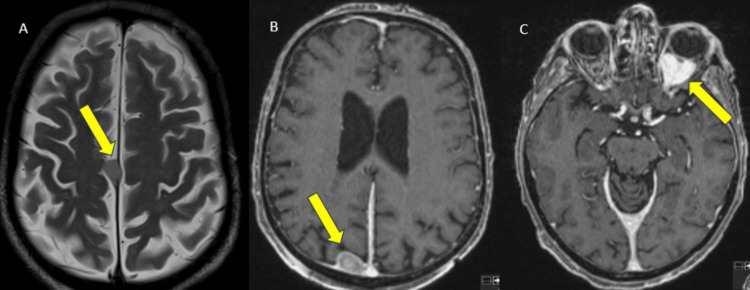
Axial T2-weighted imaging (WI) (A) and T1 fat-saturated post-gadolinium (T1FS Gd) (B and C) MRI of the brain depicts extra-axial dural-based lesions attached to the mid one-third of the falx cerebri (A, yellow arrow) and another in the right posterior parasagittal parietal convexity (B, yellow arrow). Left intraorbital, intraconal lesion is seen in C (yellow arrow). The lesions demonstrated avid enhancement (seen in B and C) MRI: magnetic resonance imaging

Subsequent investigation performed with brain MR further revealed two extra-axial dural-based lesions, one in the right frontal convexity attached to the mid-one-third of the falx cerebri, and another in the right posterior parasagittal parietal convexity, as well as a left intraorbital intraconal mass with centrifugal growth from the optic perineural region (Figure 4).

**Figure 5:**
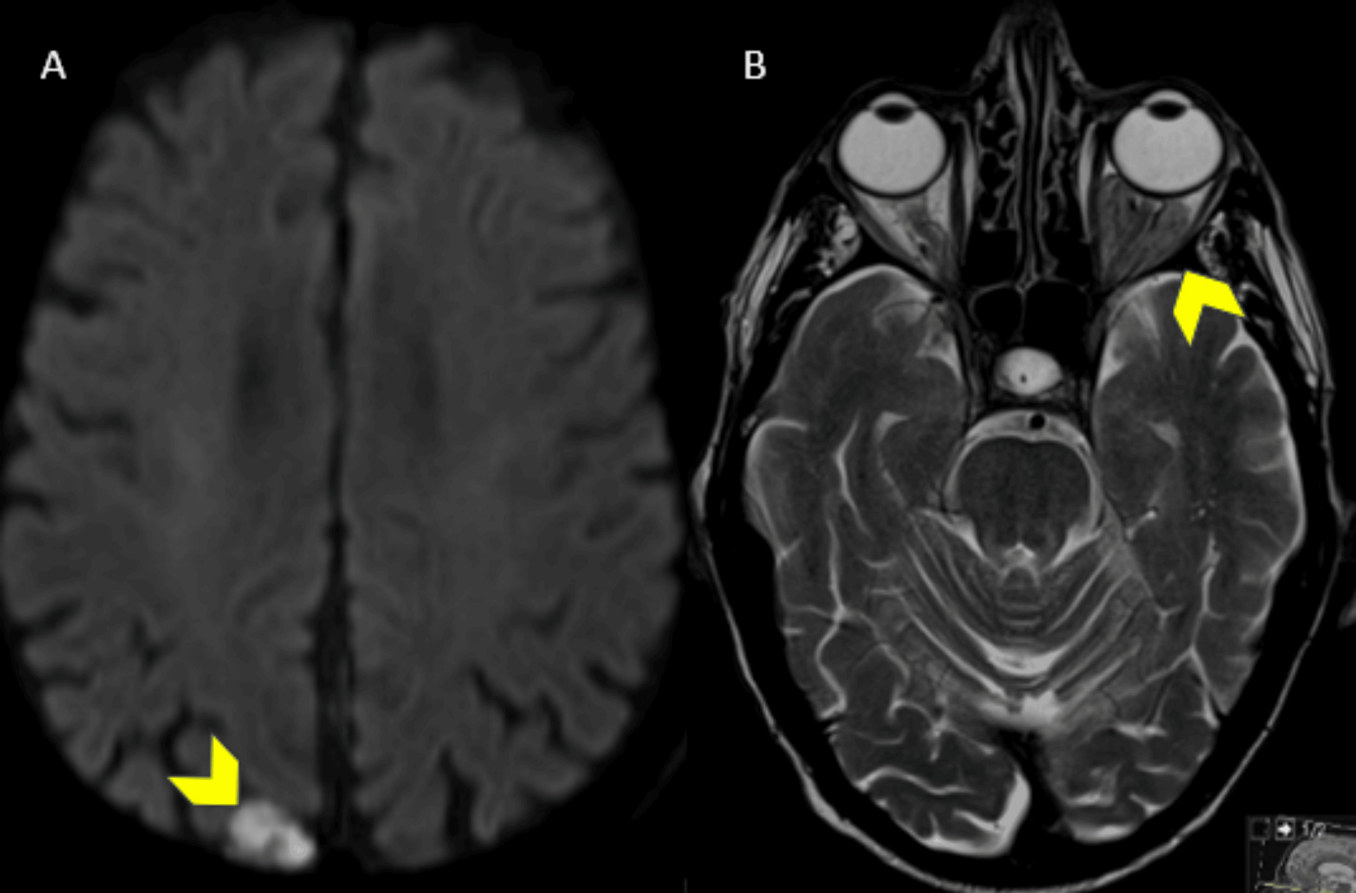
Axial diffusion-WI (A-DWI) and T2-WI MRI shows restriction of the lesions (arrowhead in A) and low T2 signal (arrowhead in B), which demonstrates their high cellularity MRI: magnetic resonance imaging

These masses demonstrated signs of high cellularity as shown by T2 iso/hypointense signal, diffusion restriction (Figure 5), and avid homogeneous contrast enhancement (Figure 4).

**Figure 6:**
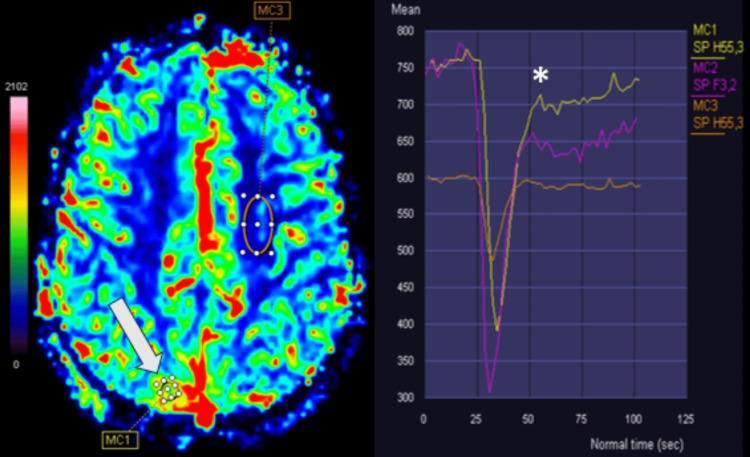
Dynamic perfusion MRI study of the lesion in the right posterior parasagittal parietal convexity (white arrow) shows lower-than-expected rCBV (3.6) and intensity time curve with partial return to baseline (asterisk) with a percentage signal recovery of 86%. All these features were not compatible with the predictable values for a meningioma MRI: magnetic resonance imaging; rCBV: relative cerebral blood volume

MRI with dynamic-susceptibility-contrast perfusion-weighted-imaging (DSC-PWI) revealed lower-than-expected relative cerebral blood volume (rCBV: 3.6) and an intensity time curve with a partial return to baseline with a percentage signal recovery (PSR) of 86% (Figure 6).

**Figure 7:**
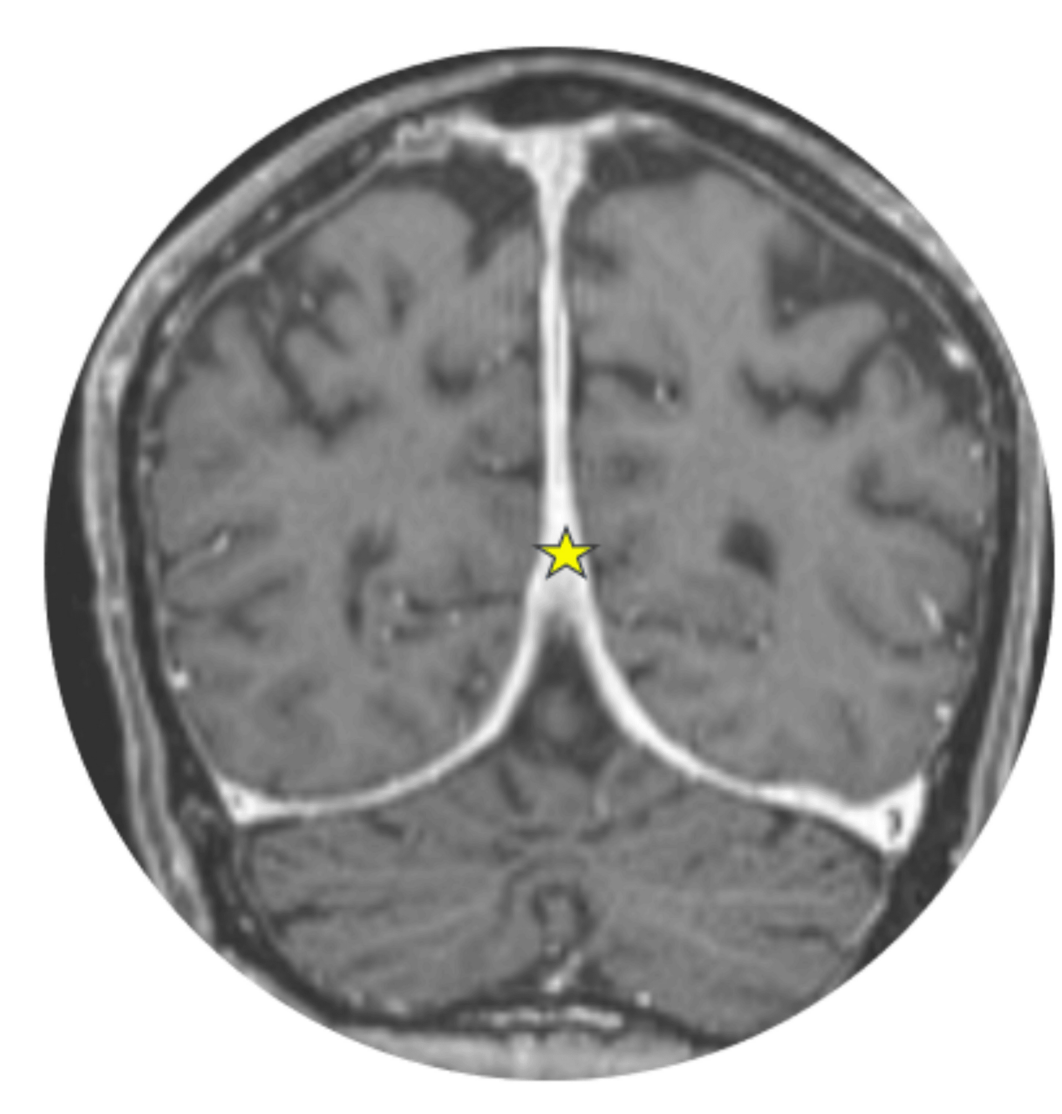
Coronal T1FS Gd MRI shows diffuse thick pachymeningeal enhancement (yellow star) MRI: magnetic resonance imaging; T1FS Gd: T1 fat-saturated post-gadolinium

**Figure 8:**
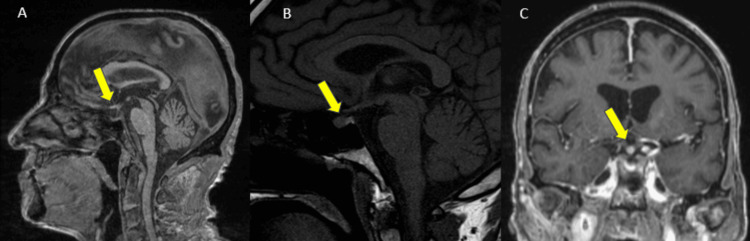
Sagittal T1 MRI demonstrates lack of pituitary bright spot (A), compared to a normal patient (B, yellow arrows). Coronal T1FS Gd (C) also shows focal nodular enhancement of the pituitary stalk (yellow arrow) MRI: magnetic resonance imaging; T1FS Gd: T1 fat-saturated post-gadolinium

Furthermore, there was diffuse thick pachymeningeal enhancement (Figure 7), focal nodular enhancement of the proximal pituitary stalk and hypothalamus, and absent posterior pituitary bright spot on T1-WI (Figure 8). No parenchymal abnormalities were noted.

**Figure 9:**
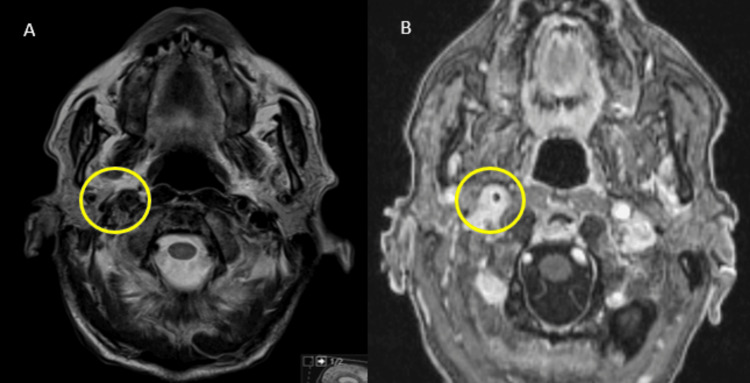
Axial T2 (A) and T1FS Gd (B) MRI denotes hypercellular infiltration with avid enhancement (B) of the right carotid sheath (yellow circles), findings similar to those in the thoracoabdominal aorta MRI: magnetic resonance imaging; T1FS Gd: T1 fat-saturated post-gadolinium

Additionally, there was hypercellular infiltration of the right carotid sheath, demonstrating T2 hypointensity and contrast enhancement (Figure 9), similar to the abdominal aortic coating found on abdominal CT.

These findings altogether pointed towards a hematological malignancy.

Surgical management

The patient underwent an abdominal biopsy of the perirenal fat/fascia in our department by Interventional Radiology.

Histopathology and molecular results

**Figure 10:**
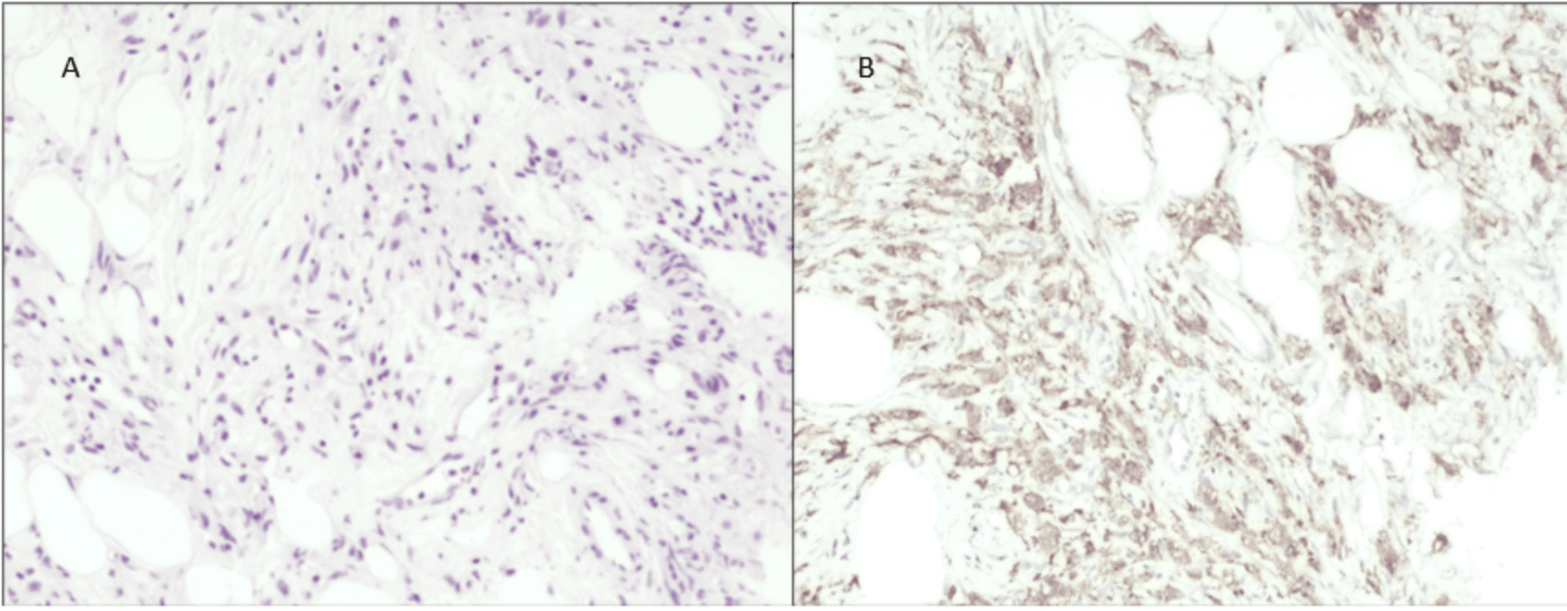
Histopathological examination of the biopsy specimen from the patient’s perirenal fascia demonstrasting abundant lymphohystiocytic infiltration (A) with positive staining for CD68 (B)

Histopathological examination of a biopsy specimen from the patient’s perirenal fascia demonstrated abundant lymphohistiocytic infiltration with positive immunohistochemical staining for CD68 and negative for CD1a and S100 (Figure 10). A complementary genetic study demonstrated the BRAF-V600E mutation, confirming the diagnosis of ECD.

Patient referral 

**Figure 11:**
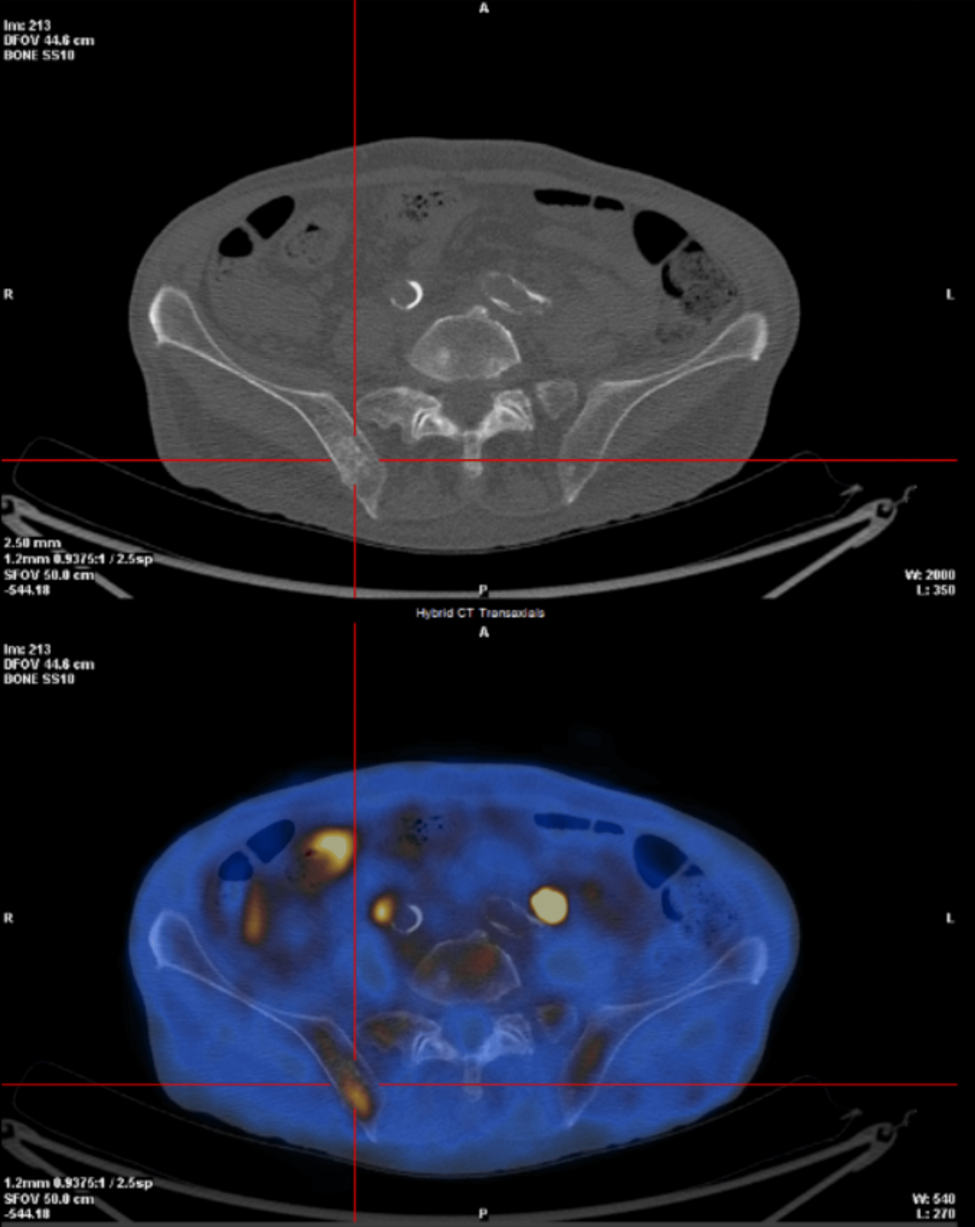
Whole body 18F-FDG PET-CT showing metabolic activity within the osteosclerosis in the right iliac bone FDG PET-CT: fluorodeoxyglucose positron emission tomography-computed tomography

**Figure 12:**
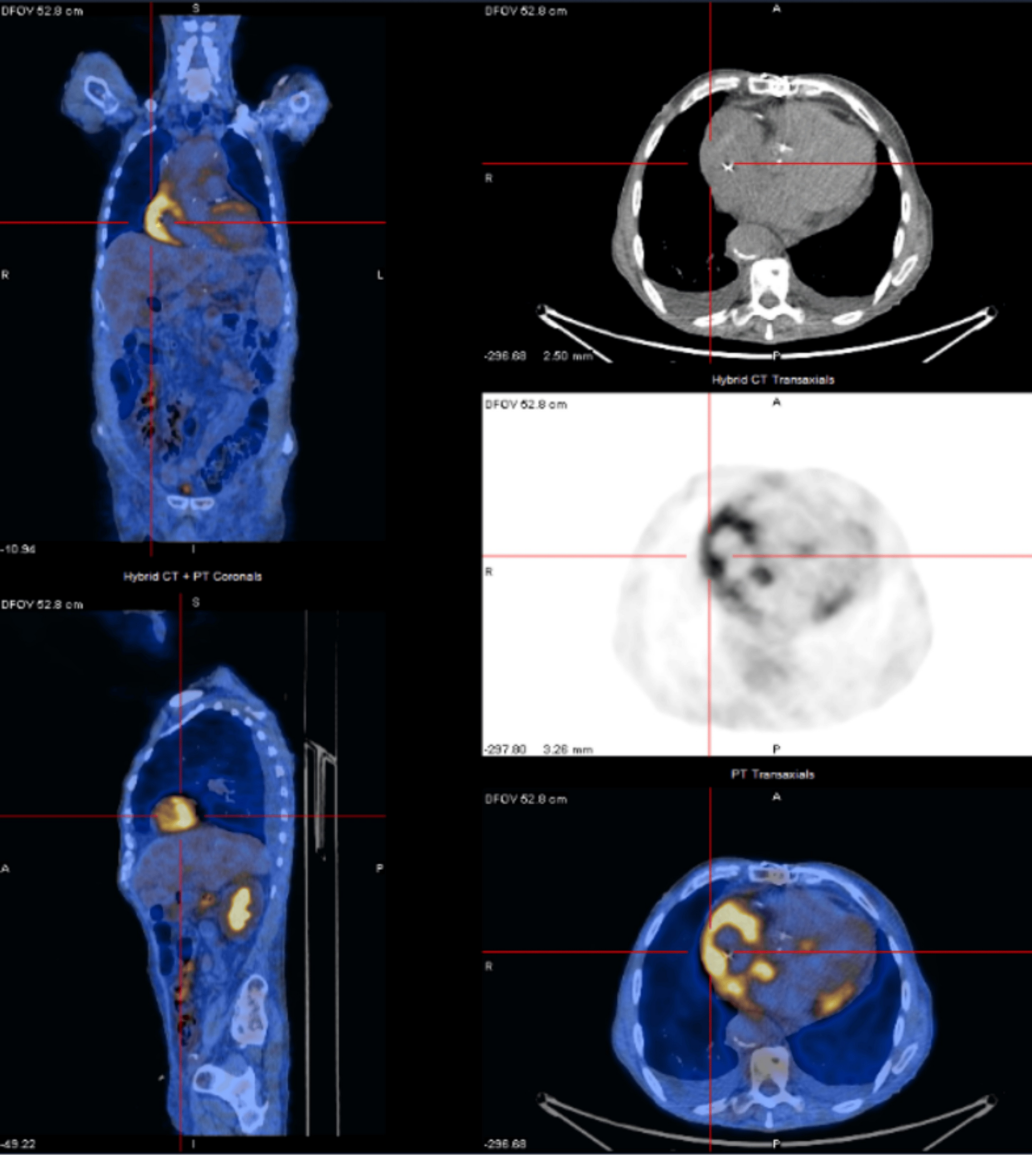
Whole body 18F-FDG PET-CT revealing increased FDG uptake in the myocardium of the right atrium, affected by the disease FDG PET-CT: fluorodeoxyglucose positron emission tomography-computed tomography

The patient was subsequently referred to the Hemato-Oncology department for specialized consultation and tailored treatment. Subsequently, a whole-body FDG PET/CT was performed, showing high metabolic activity of the intra-axial intracranial and intra-orbital masses as well as increased uptake of the aforementioned skeletal and perivascular soft tissue infiltration (Figure 11). Additionally, it revealed an increased FDG uptake in the myocardium of the right atrium (Figure 12), owing to its high metabolic activity, but without a pseudomass formation. No pleuroparenchymal manifestations were observed in the lungs.

## Discussion

There is a plethora of clinical and imaging manifestations of ECD with multiorgan/system involvement presenting with bone pain, diabetes insipidus, and/or retroperitoneal infiltration [1]. Our patient had several typical ECD manifestations, including retroperitoneal, skeletal, vascular, cardiac, orbital, and CNS involvement. This case showcases the most common findings in ECD and highlights the role of brain and body radiology in putting together isolated imaging findings while raising clinical suspicion of this rare entity, which can cause clinical misinterpretation and delay in diagnosis [12]. One of the initial diagnostic challenges in this case was the misinterpretation of intracranial lesions as meningiomas. This is a well-documented difficulty in ECD, as it can mimic other neoplasms on imaging [2,8]. Simultaneous dural, orbital, and hypothalamic involvement should raise suspicion of ECD. Furthermore, signs of hypercellularity and quantitative perfusion studies may play a role in differentiating ECD neoplasms from meningiomas, as meningiomas usually demonstrate elevated perfusion (mean rCBV: 5-12) and a strong signal drop with no tendency to return to baseline [13], in contrast with this case.

Histiocytic infiltration of the pituitary stalk correlates with clinical symptoms of polyuria and polydipsia, suggestive of diabetes insipidus, as supported by the loss of normal posterior pituitary bright spot on T1-weighted imaging (indicative of normal vasopressin storage) [14]. Although histiocytic infiltration of the infundibulum is the preeminent CNS feature of Langerhans cell histiocytosis, it usually affects male children and manifests with sharp geographic lytic skull lesions with beveled edges [15]. Sarcoid involvement of the infundibulum is uncommon in the absence of lymphadenopathy and pulmonary or mediastinal disease, and meningeal involvement is typically leptomeningeal and/or around cranial nerves [16].

Regarding abdominal findings, the absence of large abdominal lymphadenopathies is rare in lymphomas but a common feature of ECD [1]. Additionally, the circumferential coating of the abdominal aorta, helped to narrow down the differential diagnosis, since other lymphoproliferative neoplasms like IgG4-related disease tend to spare the posterior aortic wall [1,10]. Although intracranial IgG4-related disease can manifest as hypertrophic pachymeningitis with or without hypophysitis, orbital disease most commonly causes bilateral enlargement of the lacrimal gland and/or extraocular muscles, most prominently the lateral rectus muscle. Notwithstanding, head and neck manifestations are more common than hypertrophic pachymeningitis, with salivary gland involvement as the main manifestation [17].

Rosai-Dorfman disease, another rare non-Langerhans histiocytosis, differs from ECD as it presents with massive lymphadenopathy and extranodal soft tissue masses, both often in the head and neck, particularly the sinuses. Although dural-based lesions can be present in rare cases, orbital masses typically involve the lacrimal gland and/or eyelid and bone involvement is usually lytic in nature. Histopathology typically shows emperipolesis (in which histiocytes phagocytize lymphocytes, plasma cells, erythrocytes, or polymorphonuclear leukocytes). Although CD68-positive, Rosai-Dorfman is also S100-positive, which also aids in the differential with ECD [18].

ECD causes bilateral cortical sclerosis affecting the appendicular skeleton, especially in diaphysis and metaphysis of long bones like the femur, tibia, fibula, and particularly around the knees, the latter being virtually pathognomic for ECD [12,19]. Our patient had symmetric osteosclerosis in the iliac wings and proximal femoral, which also helped with the diagnosis. Cardiac involvement is a common feature in ECD, often manifesting as infiltration of the right atrium (as seen in this case), atrioventricular sulcus, and pericardium, often forming pseudotumors or causing pericardial effusion [1]. In some cases, this can lead to significant cardiac symptoms such as arrhythmias, heart failure, or pericardial constriction [8]. However, in our patient, a cardiology consultation revealed that the right atrial involvement did not result in any specific symptoms or functional impairment, despite the typical ECD-related findings.

This plethora of differential diagnoses illustrates the importance of an interdisciplinary approach in the treatment of this condition, in order to integrate the different multisystemic findings, choose the best approach for tissue diagnosis, as well as for making the right decisions related to patient referral, pharmacological management, and follow-up. Multisystemic disease involvement also entails a myriad of possible multiorgan-related complications, which again warrants a multidisciplinary team. CNS and cardiac complications may particularly impact the prognosis. 

In our case, histopathological examination confirmed the diagnosis through the identification of CD68+ CD1a- S100- histiocytes, and genetic analysis documented BRAF mutation on the same biopsy specimen. This is pivotal in all ECD patients, even if it requires an additional biopsy, as these bear major implications for therapy with BRAF inhibitors. Therapy is recommended at diagnosis in all patients, except for those with minimally symptomatic disease [20]. BRAF inhibitors are the mainstay of treatment in BRAF-positive patients as they positively impact prognosis and symptom management with quick and sustained response rates (91% according to the LOVE study). Dabrafenib has a slightly more favorable side-effect profile when compared to vemurafenib [6]. 18FDG-PET/CT scans can be used to monitor disease activity and for patient follow-up during and after treatment [6].

## Conclusions

ECD is a multiorgan histiocytic neoplasm that can present insidiously. We discussed a case of an 85-year-old male with retroperitoneal, vascular, cardiac, skeletal, orbital, and CNS involvement. Integrative brain and body radiology is pivotal for an accurate diagnosis and patient management. Meningiomas with lower-than-expected values on quantitative perfusion studies should raise suspicion of alternative diagnoses, i.e., ECD, especially in the presence of simultaneous dural, orbital, and hypothalamic involvement or other findings such as perivascular or perirenal coating and bone sclerosis. Diagnosis and management require an interdisciplinary approach with the integration of clinical findings, radiological suspicion, and histopathological confirmation.
